# Decoding Rocks: An Assessment of Geomaterial Microstructure Using X-ray Microtomography, Image Analysis and Multivariate Statistics

**DOI:** 10.3390/ma14123266

**Published:** 2021-06-13

**Authors:** Piotr Jan Strzelecki, Anna Świerczewska, Katarzyna Kopczewska, Adam Fheed, Jacek Tarasiuk, Sebastian Wroński

**Affiliations:** 1 Faculty of Geology, Geophysics and Environmental Protection, AGH University of Science and Technology, al. Mickiewicza 30, 30-059 Kraków, Poland; swiercze@agh.edu.pl (A.Ś.); fheed@agh.edu.pl (A.F.); 2 Faculty of Economic Sciences, University of Warsaw, ul. Długa 44/50, 00-241 Warsaw, Poland; kkopczewska@wne.uw.edu.pl; 3 Faculty of Physics and Applied Computer Science, AGH University of Science and Technology, al. Mickiewicza 30, 30-059 Kraków, Poland; tarasiuk@agh.edu.pl (J.T.); wronski@fis.agh.edu.pl (S.W.)

**Keywords:** microstructure, sandstone, deformation, X-ray microtomography, multivariate analysis, cluster analysis

## Abstract

An understanding of the microstructure of geomaterials such as rocks is fundamental in the evaluation of their functional properties, as well as the decryption of their geological history. We present a semi-automated statistical protocol for a complex 3D characterization of the microstructure of granular materials, including the clustering of grains and a description of their chemical composition, size, shape, and spatial properties with 44 unique parameters. The approach consists of an X-ray microtomographic image processing procedure, followed by measurements using image analysis and statistical multivariate analysis of its results utilizing freeware and widely available software. The statistical approach proposed was tested out on a sandstone sample with hidden and localized deformational microstructures. The grains were clustered into distinctive groups covering different compositional and geometrical features of the sample’s granular framework. The grains are pervasively and evenly distributed within the analysed sample. The spatial arrangement of grains in particular clusters is well organized and shows a directional trend referring to both microstructures. The methodological approach can be applied to any other rock type and enables the tracking of microstructural trends in grains arrangement.

## 1. Introduction

Rocks are the most widespread material on Earth, a source of mineral resources and host to energy resources. Their properties are strongly related to their composition and microstructure, which constitutes the spatial and geometric configuration of all rock-forming components. Therefore, a full microstructural characterization of granular materials such as rocks requires a description of grain properties, including mineral composition, size, shape and their spatial arrangement [1]. Microstructural analysis is one of the most fundamental examinations in geological sciences as it provides information on conditions of the formation, deformation and alteration processes of rocks. Acquiring such information, in turn, helps to explain the variability of functional properties of geomaterials, including their mechanical or petrophysical parameters and allows for their detailed evaluation.

X-ray microtomography (micro-CT) followed by image analysis has unlocked an emerging possibility for complex 3D material microstructure characterization [2,3,4]. However, it provides a tremendous amount of information encoded in numerical data as a result. Therefore, the extraction of viable information usually requires the application of exploratory and multivariate data analysis. By its application, one can observe patterns, trends, and clusters in grains distribution that can reflect, for instance, their origin [5].

Geoscience awaits the advent of rapid algorithms capable of analysing the mineral composition of rocks [6] and corresponding geometry of rock-forming components [7,8]. In all cases, the intention behind developing new solutions is to enhance the reproducibility of the research, minimize subjectivity and reduce time and costs [9]. One of the fast-growing in popularity platforms, R statistical software, has aroused interest especially among academics [10,11], guaranteeing the full reproducibility of the research.

As rocks tend to be highly heterogeneous, manual interpretation of their microstructural parameters would be inefficient, and automated procedures are required. In this contribution, we present a statistical characterization protocol for the microstructural assessment of geomaterials with the fully reproducible code included. The workflow was tested on a naturally deformed sandstone sample. The sample contains two deformational micro-structures i.e., the localized fault (shear fracture) with prominent surface and imperceptible macroscopically compaction bands. The study aimed to detect the microstructural changes associated with the microstructures. The protocol consists of data processing and analysis with the use of widely available freeware software of ImageJ for image analysis and the R software for numeric data analysis. It consists of micro-CT images pre-processing, feature extraction and segmentation, measurement of extracted objects and multivariate statistical analysis of the obtained results enabling the microstructural evaluation of the sample.

## 2. Materials

As a test geomaterial, a Carpathian sandstone was used. Carpathian sandstones are popular rock materials, typically used as aggregates, cladding material, and armour stone e.g., [12]. Moreover, Carpathian sandstones serve as regional reservoir rocks, hosting oil and gas deposits [13]. The Carpathian sandstones are characterized by strong internal changeability, which also directly influences e.g., the spread of geomechanical properties [12]. Moreover, the primary microstructure of the sandstones is often overprinted by diagenetic e.g., [14,15,16] and tectonic processes e.g., [17].

**Figure 1:**
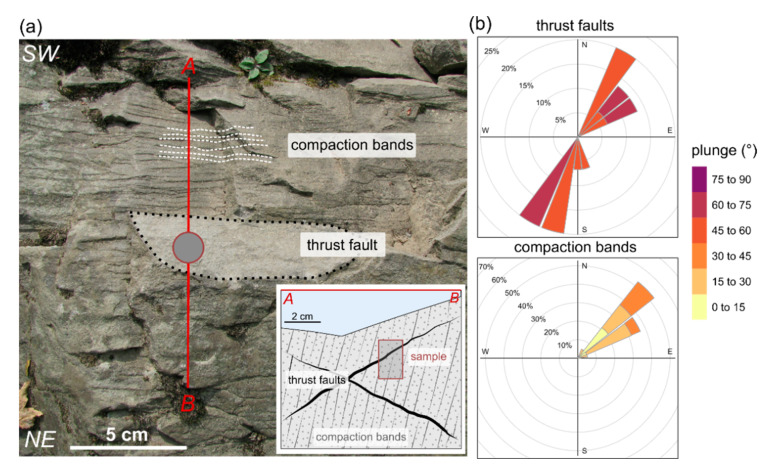
(**a**) A close view at the sandstones in the exposure and deformational microstructures within them. A situational cross-section of the sampling is presented in the bottom right corner; (**b**) The orientation of the micro-structures. Note that the orientation of normals to the micro-structures planes is presented.

The studied sample was collected at the Nasiczne locality (N49.1732, E22.5980) from a natural exposure in SE Poland. At the exposure, thick-bedded sandstones (the Otryt Sandstone) of the Krosno Beds show pervasively distributed deformation of tectonic micro-structures [18]. The micro-structures occur abundantly in the form of thrust faults and compaction bands (Figure 1a). The thrust faults dominantly dip towards NE (ca. 30°) and SE (ca. 210°) under low to moderate angles (15–45°). The compaction bands dip towards SW (ca. 230°) under moderate to high angles (55–85°; Figure 1b). The thrust faults form distinct surfaces, whereas the compaction bands are sometimes macroscopically imperceptible.

The centimetre-scale samples were collected for microscopical and petrographical analyses. The additional cylindrical core sample of approximately 2 cm in diameter and 2 cm in height bearing the tectonic micro-structures was withdrawn for microstructural assessment with the use of X-Ray microtomography (Figure 1a). This sample was collected with a reference to geographical directions, allowing to reconstruct the original position of the sample after scanning and correlate observed deformation micro-structures in the exposure with the microstructure of the scanned sample.

## 3. Methods

**Figure 2:**
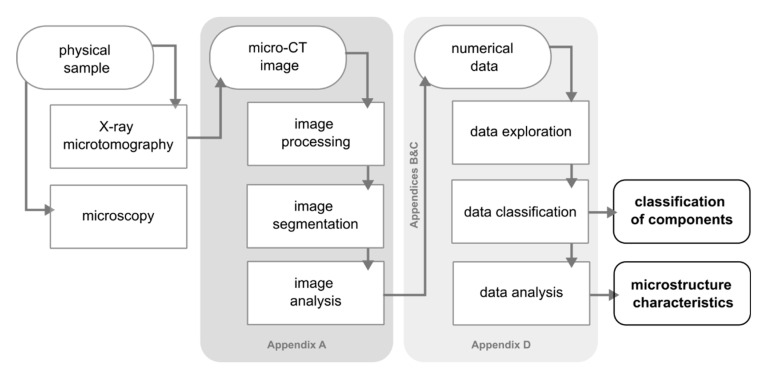
The workflow of data processing and analysis.

The methodology consists of two main techniques (Figure 2). The first one comprises image processing and analysis and the second one concerns processing and statistical analysis of the resultant numerical data obtained by the former. The protocol and reproducible code described in detail are available in Appendix A, Appendix B, Appendix C and Appendix D. The analysis was supplemented by the previous examination of the specimen using polarized light microscopy and cathodoluminescence. The processing and analysis were performed on a notebook equipped with an Intel Core i5-7300HQ (Intel Corporation, Santa Clara, CA, USA) CPU, NVIDIA GeForce GTX 1050 (Nvidia Corporation, Santa Clara, CA, USA) GPU and 16 GB of RAM operating on Windows 10 (64-bit version). The validation of the protocol and calculations of grains ordination on a standard sample was performed (see Appendix E).

### 3.1. Polarized Light Microscopy and Cathodoluminescence

Supplementary analysis of thin sections corresponding to the core sample was performed. Basic mineralogical composition of the sample was recognised using the point count method with 300 points per thin section utilised. The analysis and microphotographs were taken using an AxioImager.A1m microscope (Carl Zeiss Jena GmbH, Jena, Germany) in both plane- and cross-polarised light. Additionally, petrographic studies using an Eclipse Ci-L microscope (Nikon Instruments Inc., Melville, NY, USA) with a Mk5 cold cathode (Cambridge Image Technology Ltd., Hatfield, UK) under 15 keV voltage with the electron beam current of 400 µA were performed. The polarized light microphotographs, as well as cathodoluminescence photos, were taken at the Department of Fossil Fuels, AGH University of Science and Technology (Kraków, Poland).

### 3.2. X-ray Microtomography

The collected cylindrical sample was subjected to X-ray computed microtomography at the Laboratory of Micro and Nano Tomography of the AGH University of Science and Technology. The sample was scanned with a Nanotom S device (GE Sensing & Inspection Technologies GmbH, Wunstorf, Germany) equipped with a nanofocus X-ray tube. The tomograms were registered on a HAM C 7942CA-02 detector (Hamamatsu Photonics K.K., Hamamatsu, Japan). The reconstructions of the measured objects were performed with an aid of the proprietary GE software datosX ver. 2.1.0 and using the Feldkamp algorithm for cone-beam X-ray CT [19]. The sample was scanned at 100 kV of source voltage and 400 μA tube current, with a full, 360° rotation of the specimen in 1800 steps. The exposure time equalled 500 ms and the frame averaging of 5 and the image skip of 1 was applied, resulting in a scanning time of 85 min. A stack of images contained approximately 2000 slices of 2300 × 2300 pixel-sized images. The resolution of the image stack was 12 µm. The resultant 3D image of the sample represented in grey-scale reflects the X-ray attenuation, which is related to the density (ρ) and atomic number (Z) of the sample-forming components e.g., [20]. The coefficient of X-ray attenuation is proportional to ρ and Z^4^ at low scanning energies such as these utilized in the present study e.g., [21]. The components with the lowest coefficient of X-ray attenuation are represented by the darkest pixels, while the brightest ones represent those of the highest attenuation e.g., [21].

### 3.3. Image Processing and Analysis

**Figure A1:**
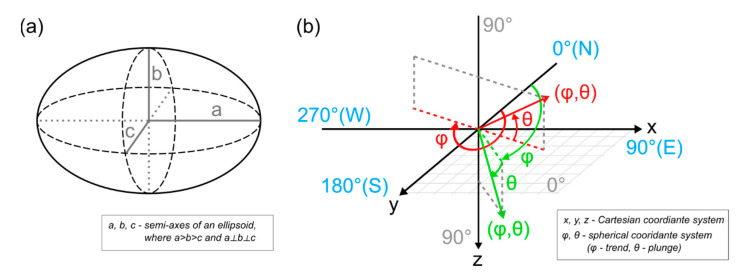
(**a**) An ellipsoid and dependency between its semi-axes; (**b**) The rule of coordinates transformation from the Cartesian coordinate system in Fiji software to the spherical coordinate system of vectors representing semi-axes of the ellipsoids.

The image processing and analysis were performed using the Fiji software (ImageJ version 1.53c, Java 1.8.0_66, 64-bit) [22]. The procedure of the processing and analysis of the image is described in detail with a sequence of steps to perform in Fiji in Appendix A. Firstly, the raw image (2284 × 2284 × 1674 pixels) was converted into the 8-bit format resulting in a file size of 8 GB. Subsequently, image enhancement was performed (Appendix A, step 5). Consequently, corrections of brightness and contrast were applied, improving the legibility of the image. Considering the cylindrical shape of the sample, the image was cropped and limited to 1000 pixels (12 mm)-sided cube constituting a volume of interest (VOI; file size 0.9 GB). On the VOI correction of the X-ray attenuation using BaSiC [23] was applied. Feature extraction was performed using the global thresholding method [24]. The granular components of the rock above the global threshold value were extracted and binarized. The binarized image was filtered using a 3D median filter (kernel size 2 × 2 × 2 pixels) and the objects at the image edges were removed. Lastly, a size filter, removing the objects below 100 voxels in volume (approximately <0.07 mm in diameter), was applied to eliminate the artefacts and imprint of the smallest insignificant objects with the size closest to the resolution of the image. Finally, the resultant image was segmented to attribute individual objects with a unique label. The total time of image processing was approximately 30 min. The prepared image was subjected to the measurements. They were performed using the ImageJ 3D suite [25] and MorphoLibJ [26] plugins and lasted approx. 40 min in total. The results of these measurements were combined and a data set consisting of 44 parameters was prepared using R (Appendix B). The parameters were prepared based on these available by default in the image analysis software. Their choice was dictated by the simplicity of their interpretation as well as their relatively short computation time. The parameters fall into 4 categories describing the chemical composition, size, shape, and spatial orientation and arrangement of each object representing a grain. Each category is represented by several unique parameters (Appendix C, Table A1). By introducing a higher number of parameters i.e., 44, one obtains a possibility of tracing detailed variability of grains properties is provided. Parameters concerning the spatial orientation of grains were transformed into a spherical coordinate system (Appendix C, Figure A1). The calculated parameters are input variables for further analyses which were performed in R software with additional libraries (Appendix C, Table A2).

### 3.4. Exploratory Data Analysis

For exploration and visualization of the data set, principal component analysis (PCA) [27,28,29] and factor analysis (FA) [30] were performed. PCA and FA are statistical techniques used for multivariate data exploration and allow for dimension reduction, simultaneously retaining most of the variation in the data set. As a result of PCA, principal components (PCs) are calculated which are the eigenvalues and eigenvectors of the correlation matrix of the original variables. PCs are mutually uncorrelated and can delineate different characteristic features of the data set objects. Each principal component (PC) explains a portion of the total variance in a data set. Because variables are measured in a mixture of units and ranges, the data were standardized. To determine the number of PCs, a scree plot [31] was used. To uncover the underlying structure of PCs, FA was performed. The structure of PCs was examined using standardized loadings and indexes of uniqueness, communality and complexity [32] (Appendix D, step 4).

### 3.5. Cluster Analysis

Cluster analysis is a method of unsupervised learning and pattern recognition that allows for classifying and identifying groups in a data set. Cluster analysis allows similar observations to be grouped into homogenous subsets with no previously known pattern. The data is grouped based on the similarity between the observations concerning the values of variables for each observation. The distance between the pair of observations is a measure of the dissimilarity. Observations within the same cluster are similar and different from other clusters. The clusters themselves are dissimilar. In the studied case, cluster analysis based on a k-medoid method using the Clustering Large Applications (CLARA) algorithm [33,34] was performed. The CLARA algorithm is a variant of the Partitioning Around Medoids (PAM) algorithm dedicated to large data sets. Clustering was performed on the standardized original data set using the Manhattan distance matrix. For the classification of the components, the variables describing properties that are independent of the spatial orientation of components, i.e., these describing their chemical properties, size and shape were employed. The optimal number of clusters was determined based on silhouette statistics [35] (Appendix D, step 5).

### 3.6. Microstructure Assessment

Microstructure assessment was performed based on the results of the cluster analysis. The distribution of parameters was examined in the previously determined groups. The distribution of variables describing the chemical composition, size, and shape of grains were illustrated on violin plots cf. [36]. For the variables describing the spatial configuration of objects, the radial distribution function was calculated [37]. The radial distribution function finds the centre of grain in a given position at a radial distance from the centre of a reference sphere, which was set to the centre of the VOI. This measurement characterizes packing structures and contains information about interparticle correlations and their organization. To examine the spatial orientation of grains, rose plots were constructed. The rose plots provide information on the direction and dip angle of particular grains. The orientation of the grains was projected based on the orientation of fitted ellipsoid semi-axes (Appendix D, step 6).

## 4. Results

### 4.1. Sample Characterization

**Figure 3:**
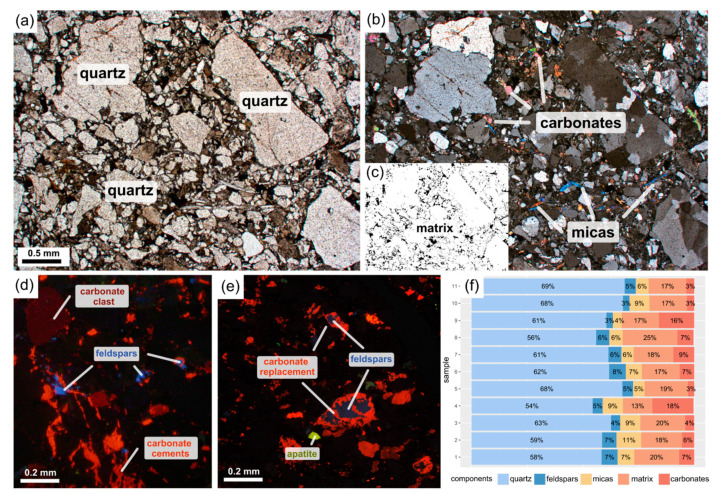
(**a**) Microphotographs showing rock composition and microstructure corresponding to the sampled rock shown in the plane-polarised light and (**b**) cross-plane polarized light; (**c**) A mask of the matrix extracted from Figure 3a; (**d**,**e**) the cathodoluminescence microphotographs showing the mineral composition of the sample; (**f**) Results of the mineral composition determination using the point-count method on the corresponding thin sections.

The test material is a poorly sorted sandstone. It is composed predominantly of quartz grains (54–69%), subordinately of micas (4–11%), including a substantial amount of muscovite and a subordinate share of biotite, feldspars (3–7%), and carbonates (3–18%) in the form of grains and cement (Figure 3a–f). The last mentioned sometimes replace feldspars (Figure 3d–f). The intergranular space of the sandstone is filled with matrix (Figure 3c), composed of a mixture of silt-sized minerals of the granular framework and clay minerals. The matrix constitutes 13–25% of the rock’s volume (Figure 3a–f). Other accessory minerals occur in a small proportion (<1%) and are represented by heavy minerals such as pyrite or apatite.

**Figure 4:**
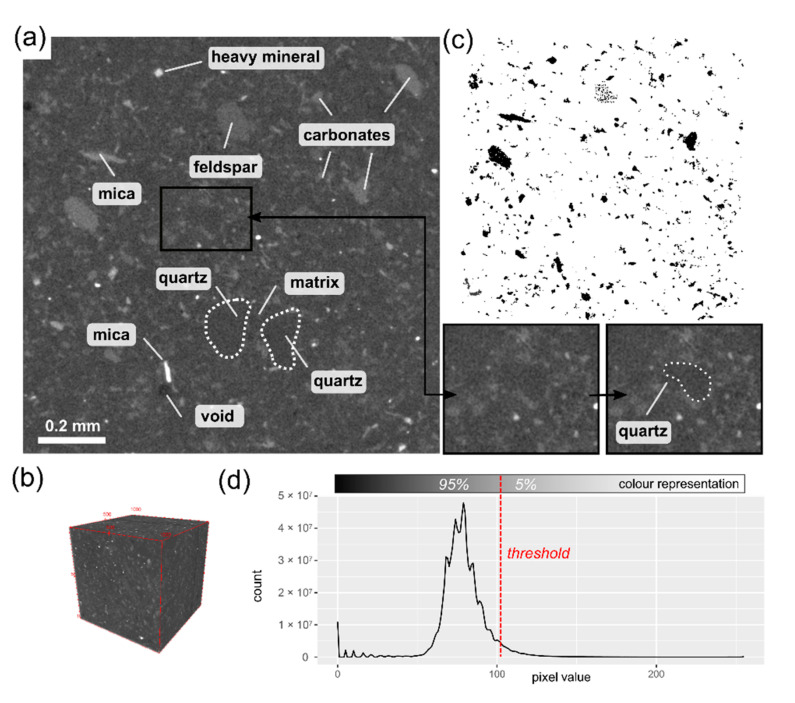
(**a**) Selected micro-CT image of the sample with mineral components marked. Note that outlines of quartz grains are visible; (**b**) 3D visualization of the VOI; (**c**) The extracted and analysed mineral components, the image corresponds to this shown in Figure 4a; (**d**) Histogram of colour distribution (pixel value) within the analysed volume of the sample.

In the micro-CT image, the mineral composition is harder to recognise as some minerals show similar properties in terms of X-ray absorption and consequently attain the same intensity of pixel values. Quartz, feldspars and matrix together constitute 90% of the rock’s volume and in the micro-CT and appear as grey-coloured background (Figure 4a,b). Some minerals are distinguishable from the background as they show higher absorption of X-rays and thus lighter shades of grey. These minerals represent carbonates, micas and heavy minerals, and their granular form can be extracted from the micro-CT image. These grains constitute 5% of the VOI (Figure 4c). The remaining 5% of the VOI are made up of voids, which constitute unfilled spaces within the rock. These volume shares are comparable with the results of mineral composition recognition from the point count method. The extracted components are pervasively distributed within the VOI, with a total number of 25.015 individual grains.

### 4.2. Data Exploration

**Figure 5:**
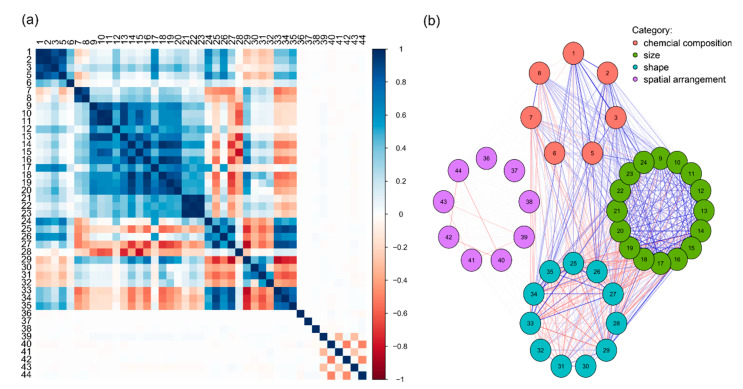
(**a**) Heatmap of the correlation matrix between the variables. The colour designates the value of the correlation coefficient between particular variables; (**b**) A graph showing correlation between variables in a form of network. The colour of the nets corresponds to this presented in Figure 5a and designates the correlation coefficient. The variables are grouped regarding their categorical subdivision. Numbers (1–44) correspond to the names of variables (see Appendix C, Table A1).

Each grain was described with 44 parameters. The minimal intensity parameter was excluded from the further analysis as its variance equals zero. The initial variables are correlated. The correlation coefficients between variables are high, absolute values above 0.5, especially in the case of variables describing similar properties within the category (Figure 5a). However, correlations between variables of a different category are also noticeable. The mutual correlation between variables is strong in the case of chemical properties, and size and shape variables. The variables in the category of spatial arrangement show either weak or no correlation, both between the variables within that category and the variables from other categories (Figure 5b).

**Figure 6:**
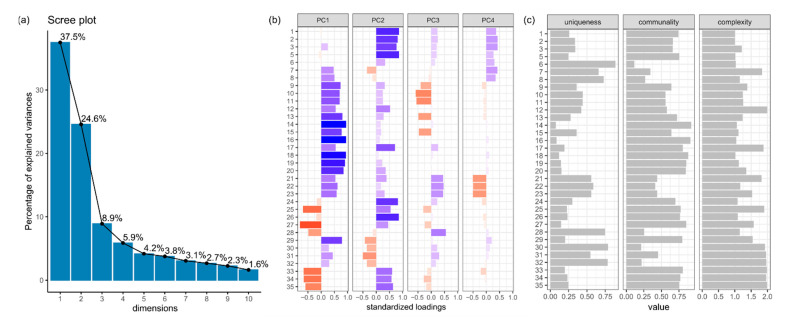
(**a**) The scree plot; (**b**) The structure of the first four PCs. Colours correspond to the value of standardized loading; (**c**) The statistics for the first two PCs. Numbers (1–35) correspond to the names of the variables (see Appendix C, Table A1).

For the correlated variables (all except the spatial arrangement category), PCA was conducted. The scree plot (Figure 6a) shows that the initial number of variables can be reduced to a lower number of dimensions. The first four principal components (dimensions) explain 76.9% of the total variance in the data set, whereas the first two account for the majority of it i.e., 37.5% and 24.6%, respectively. The first two PCs depict the general variability in the data set.

Further dimensions explain less than 5% of the variance. The structure of the first four PCs indicates an ordered structure of the initial variables underlying the dimensions. The first PC shows an input of variables describing geometrical properties of the grains such as the size and shape (Figure 6b; variables no. 9–35), whereas the second PC is loaded from the variables describing the chemical composition (Figure 6b; variables no. 1–8) and geometrical properties. However, in the case of PC2 the loading values from the geometrical variables are lower, and in PC3 and PC4 they are mainly below 0.5 of the absolute value. The communality index for the first two PCs indicates that variables such as describing intensity distribution statistics, Euler number or ratios of ellipsoid semi-axes (Figure 6c; variables no. 6–8, 28, 30–32 respectively) are more unique as they show a low proportion of common explained variance. The average complexity equals 1.4.

**Figure 7:**
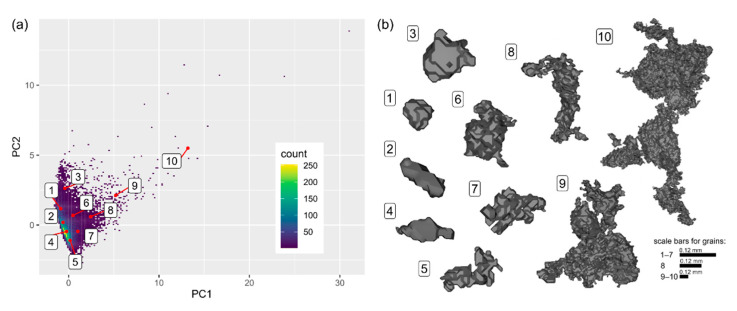
(**a**) Projection of the objects into the PC coordinate system using a density map; (**b**) The visual sample of 10 grains (objects) from the dataset. The labels correspond to these in (**a**).

The projection of the data onto the PC coordinate system suggests that the data do not show any distinctive clusters and are projected densely and continuously closer to the origin of the PC coordinate system. Towards increasing PCs, the tail of outliers is observed (Figure 7a). The outliers are represented by the largest individual grains or aggregates of mutually connected grains (Figure 7b).

### 4.3. Classification of Components

**Figure 8:**
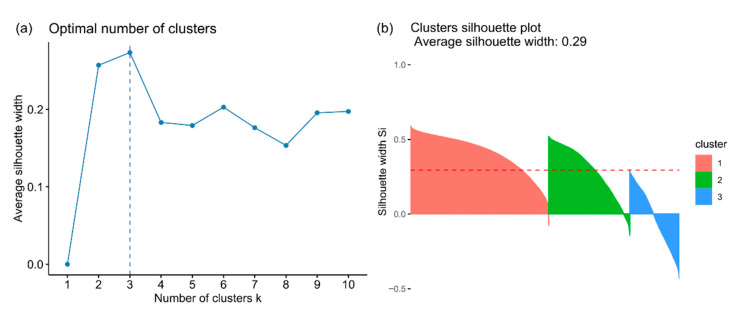
(**a**) The optimal number of clusters determined using average silhouette width statistics; (**b**) The quality of clustering using 3 as the optimal number of clusters based on average silhouette width statistics.

The average silhouette width statistics indicate 3 as the optimal number of subsets within the data (Figure 8a), with an average width of cluster equalling 0.29. The clustering of the data set returns three clusters with 12,878, 7519 and 4618 objects, respectively. The average widths of the three obtained clusters are 0.42, 0.30 and 0.01, respectively. The first two clusters show a major contribution of the positive values indicating that the objects belonging to these two clusters are similar within their cluster, whereas in the third one, a great number of negative values points on a general dissimilarity and diversification of the objects within that cluster (Figure 8b).

**Figure 9:**
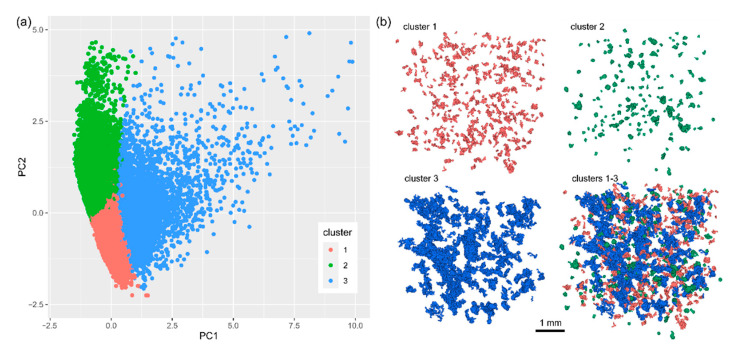
(**a**) Results of the cluster analysis: data classification in the PC coordinate system and (**b**) visual representation of the clustering results presented as a fragment of the VOI.

Observations within the first and second cluster are concentrated, whereas, in the third one, they are more dispersed. The third cluster also consists of outlier observations (Figure 9a). The results of the clustering appear to be satisfactory upon visual inspection. The first and second clusters represent relatively small grains as compared to those from the third cluster. The grains in the first cluster appear to be more irregular in comparison to these of the second cluster. The grains of these two clusters are secondary and fill the intergranular space of the specimen, corresponding to the main (largest) grains of the third cluster (Figure 9b).

### 4.4. Microstructure Assessment

**Figure 10:**
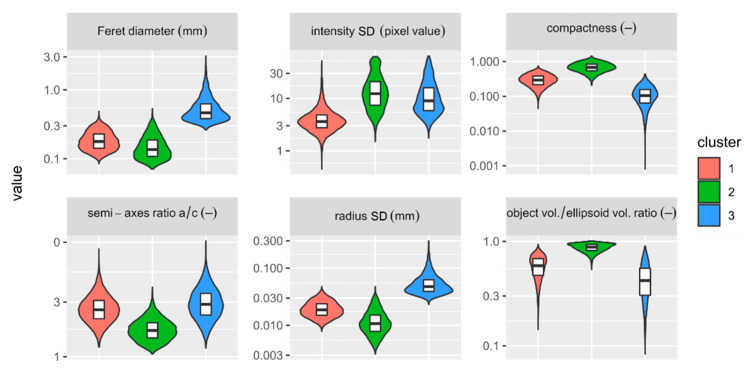
Distribution of the selected parameters in the clusters.

The general observations highlighted in the previous section find confirmation in the numeric data. The clusters show diversified grain properties (Figure 10). In general, grains in the first and second clusters are smaller in comparison to those of the third one (Figure 10; e.g., the median Feret diameter equals 0.18, 0.16, 0.46 mm, respectively). The second and third clusters are more chemically diversified in comparison to the first one (Figure 10; e.g., median intensity SD equals 13, 9, 3, respectively). The second cluster consists of regular grains of ellipsoidal and spherical shapes (Figure 10; e.g., high compactness and low semi-axes ratio a/c), whereas the first and the third ones contain more irregular grains (Figure 10; e.g., higher radius SD values and lower object to ellipsoid volume ratios).

**Figure 11:**
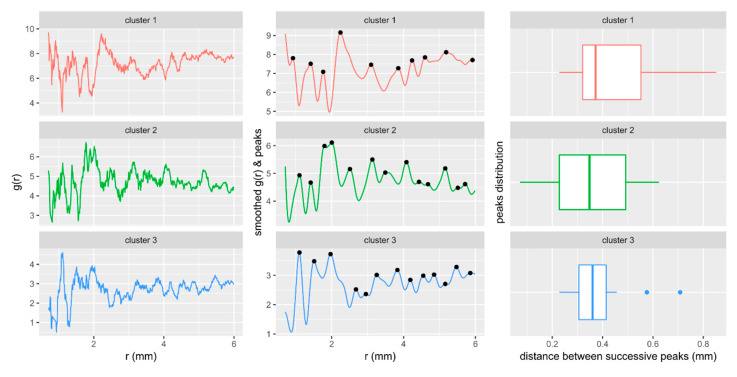
Radial distribution function g(r) calculated for the geometrical centres of grains in the clusters.

The grains are evenly distributed in the specimen. With increasing distance from the centre of the VOI, the periodicity and density of the distribution of grains’ geometrical centres remain stable (Figure 11). The specimen shows a dense and compact packing of the granular framework. The distance between the successive peaks, i.e., the distances between the geometrical centres of grains in particular clusters, corresponds to the average grain size in the third cluster. Moreover, all clusters show a similar median value which ranges from 0.35 to 0.37 mm, indicating that the third cluster played a determinative role in controlling the microstructure of the sample.

**Figure 12:**
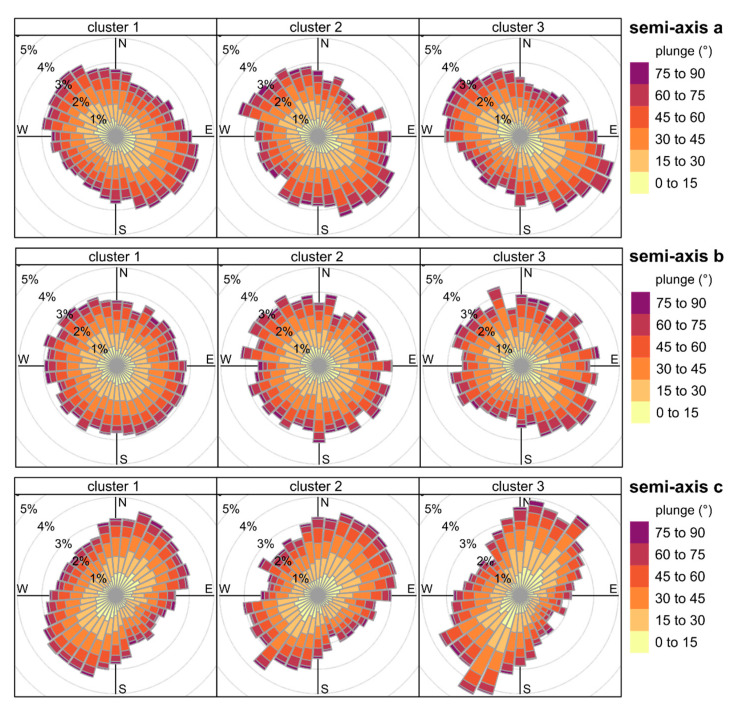
Spatial orientation of the grains in the clusters. The width of a bin is 10°; N equals 0°.

The grains are well arranged in all clusters and the distribution of their orientation delineates distinct trends, indicating the presence of directional microstructure (Figure 12). 

The distribution of the longest semi-axes (a) orientation in the grains is mainly NE-SW-oriented, whereas the orientation of the intermediate semi-axes (b) is uniform in all directions in all clusters. Conversely, the shortest semi-axes (c) are NW–SE-oriented. The number of observations in the dominant direction is higher by 1 to 3 per cent points in comparison to other directions. The orientation of the shortest semi-axes (c semi-axes), which are normal to the ab surfaces, indicates that the grains are predominantly tilted in the NE or SW direction under high angles. However, the dominant directions are slightly shifted between the clusters. The spread is best visible between the second and third cluster considering the orientation of semi-axes c. In the second cluster, the dominant direction aligns with 50°, whereas in the third cluster with 30°. It is noteworthy that within the third cluster there is a considerable number of observations trending 50° under low plunge values, as well (Figure 12; the third cluster c semi-axes orientation). In the first cluster, the dominant direction is the resultant of these two directions. The frequency of observations in the opposite directions remains at a similar level.

## 5. Discussion

### 5.1. Image Processing and Analysis

Segmentation of the image remains challenging as rock-forming minerals typically display similar attenuation properties [20]. Ideally, minerals should be characterized by a diversified density. Therefore, not all granular materials or rocks would be suitable for the analysis. Likewise, in this case, only a limited content of the whole sample was analysed. Although the selected components constitute only 5% of the total rock’s volume, the grains subjected to the analysis are widespread and dispersed across the entire VOI. Therefore, in our interpretation, they are representative and reflect the general microstructure of the sample. As a consequence, even an analysis of a limited volume can be sufficient to obtain proper information on the microstructure of a geomaterial as a whole. Some of the grains seem to be connected (Figure 7b), which may be seen as an artefact. However, as revealed by the supplementary analysis of the thin sections, carbonates may form such structures. Nonetheless, the connected grain chains are inevitable as some of them might be naturally contacting. Therefore, more advanced methods for grain segmentation could be considered cf. [21].

### 5.2. Data Exploration

The first two PCs explain 62.1% of the total variability in the data set. This value is slightly above the minimum recommended threshold of 60% [38]. In general, it is advised to maximize the value of the total explained variance retaining subsequent PCs until they account for at least 95% or to the point when PCs explain at least 5% of the variance [38]. Therefore, the first two PCs allowed for projecting the data onto a two-dimensional space and tracing the basic properties responsible for data variabilities such as grain size, shape and chemical composition. Following the mentioned rule, and adding the next two PCs would not significantly increase the explained variance. Conversely, this would complicate projecting the data by imposing four dimensions in total. The value loadings should be at least 0.3–0.4 of the absolute value. In this case, they were usually above 0.7 which indicates a strong correlation of the initial variables with the PCs [38]. Some of the variables load more than one PC (Figure 6c; complexity index), but the mean complexity index remains low (1.4 on average) and the structure remains clear. In the case of high complexity index, a rotation of the PCs to simplify the interpretability of the underlying structure can be considered cf. [29]. Usually, the projection of the data depends on the purpose of the analysis. It is a common practice to only utilise selected single variables.

### 5.3. Data Classification

In this study, a k-medoid-based clustering algorithm was chosen. The advantage of this is the following. Firstly, it minimizes the sum of dissimilarity in the pairs of observations, contrary to the k-means algorithm which minimizes the total mean standard error. The k-medoid algorithm is less sensitive to both the outliers and noise. Additionally, cluster centres are designated based on real observation (medoid) and can handle the clusters of different density and size cf. [39]. In the case of high dimensional data, the Manhattan distance matrix usage is recommended [40]. In the case of this study, the statistically optimal number of clusters was designated as three with the average silhouette width statistics equalling 0.29. The value of the last quantity indicates that the data might be structured and support the validity of cluster analysis application [34]. However, the designation of the number of clusters is subjective and depends on the method applied cf. [41]. The choice of the cluster number can be based on silhouette statistics, internal or external criteria [42]. The latter assumes a comparison of the cluster solution with external results. For such a solution, manual and visual assessment of the cluster solution (see Figure 9b) and expert knowledge should be considered for the validation of the obtained results.

Comparison of clustering results with a different number of clusters can be applied as a data exploration method to gain an understanding of the patterns and features of a data set. Initially introducing a higher number of clusters might also result in a more detailed classification of grains allowing for peculiar grain clusters detection. The choice of the initial variables (grain parameters) as input for cluster analysis is also important, as their different combination may lead to significantly different results. Therefore, their choice should be based on the purpose of the analysis and the classification of the selected aspects of data. By using only selected parameters e.g., only from one category, distinct classification of chemical composition, size or shape of grains could be obtained. Therefore, introducing a relatively high number of parameters i.e., 44 was aimed to provide as much information about the variability of the sample as possible (e.g., Figure 6c). The role and significance of particular parameters depend on the nature of geomaterial and its grains and it may vary depending on the material.

### 5.4. Microstructure Interpretation: A Case Study

The preferred arrangement of grains in sedimentary rocks such as sandstones can be either a result of sedimentary or deformational processes cf. [43]. In the case of the studied sample, their deformational origin seems to be responsible for the resultant microstructure of the sample. The grains mostly dip under moderate to high angles, indicating their rearrangement and steepening during the deformation. Moreover, the alignment of the grains is consistent with the direction of micro-faults and compaction bands. In the case of the sedimentary origin of grains arrangement, the grains are tilted under low to moderate angles and the presence of directional trends is a result of their directional transportation cf. [43]. The direction of the paleo-current inferred from the surrounding strata indicates a NE-SW-directed material transport [44]. This direction coincides with the orientation of the observed deformation, but the arrangement of grains indicates that the deformation overprinted the primary sedimentary microstructure. 

**Figure 13:**
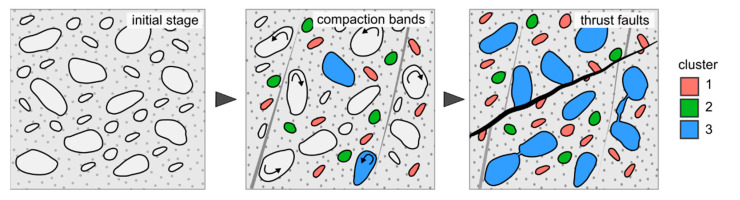
The evolution of the Krosno Beds from the Nasiczne site decoded from the studied sample.

The application of cluster analysis grouped grains into groups among which two of them are distinctive i.e., cluster no. 2 and 3. The second cluster can be related to primary grains of the rock, heavy minerals such as biotite, which were deposited during sedimentation and were involved in deformation from the beginning. Their alignment is in accordance with the compaction bands. The third cluster consists of grains of mixed origin. It seems that initially they were involved in the reorientation recorded by grains of the second cluster and then overprinted by diagenetic processes during which some of the original grains were dissolved and replaced by carbonate cement e.g., (Figure 3d,e). The carbonate cementation and the replacement of feldspars by carbonates is commonly observed in Carpathian sandstones e.g., [15,16]. The introduction of carbonate cement seems to be associated with the formation of micro-faults. Hence, the correspondence between the micro-fault orientation and the trend in cluster 3 is observed. The faulting can cause micro-fracturing of the material, thus promoting the formation of elongated voids, usually in accordance with the faulting direction. As result, a space for cement crystallisation is formed and thus the presence of mutually connected grains can be the result of this process (Figure 13).

## 6. Summary and Conclusions

The presented protocol allows for the complex 3D characterization of granular geomaterials microstructure utilizing 3D microCT images, image analysis and multivariate statistics. The protocol consists of image processing analysis and the multivariate analysis of its results. The outcome of the image analysis is a detailed description of grains with 44 unique parameters describing compositional properties, size, shape, morphology, and spatial arrangement of each grain. By the application of multivariate statistics and data exploration, an understanding of the characteristics of the grain can be obtained. Based on the parameters, cluster analysis is conducted which classifies the objects based on their similarity, allowing for characteristics of groups to be distinguished within a data set. The methodological approach can be applied to any other rock type and other geomaterials. The proposed protocol is useful in deciphering the nature of geological processes recorded in rocks, as well as being used for the explanation of the anisotropy of material properties. The detailed numerical characterization of the microstructure enables the tracking of even the gentlest trends within geomaterials. The application of freely available software for the analysis increases its availability and broadens the audience. The attached protocol with the included code can be reused in the presented form or modified by means of the implementation of proprietary solutions and thus promotes the reproducibility of the research.

The results confirm the presence of deformational microstructures in the studied sample related to both deformations. The grains were clustered into groups representing different compositional and geometrical aspects of the granular framework. With the use of supplementary microscopical studies, the origin of grains was possible to be determined and the sequence of recorded processes recognized. Firstly, the grains in the sample underwent deformation and reorientation leading to a steepening of the grains and their compact packing, with the formation of compaction bands. Subsequently, micro-faults were formed and this was associated with carbonate cement intrusion leading to the replacement of some grains and their connection, probably by micro-fracturing of the material during faulting.

Summing up, two major conclusions concerning the studied material can be presented: (1) the grains are pervasively and evenly distributed within the analysed sample and show compact packing and, (2) the spatial arrangement of grains is well organized and show directional trends referring to the orientation of compaction bands and micro-faults.

## Figures and Tables

**Figure A2:**
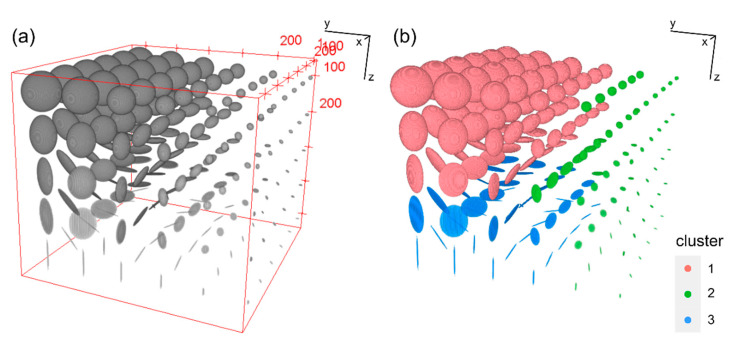
3D visualization of the standard sample (**a**) and clustered grains (**b**).

**Figure A3:**
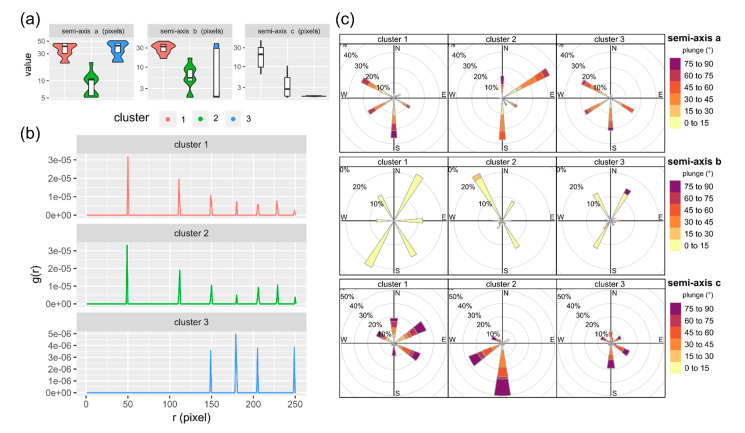
(**a**) Distribution of the selected parameters; (**b**) Radial distribution function g(r) calculated for the geometrical centres of grains; (**c**) Spatial orientation of the grains in the clusters.

## Data Availability

The data set and the codes included in appendices are also available on GitHub (https://github.com/piotrstrzelecki/materials, accessed on 5 June 2021). The other data presented in this study are available on request from the corresponding author.

## Appendix A. The Protocol of Image Processing and Analysis in Fiji

ImageJ version 1.53c Java 1.8.0_66 (64-bit)

Additional plugins required: MorphoLibJ, 3D image suite, BaSiC

Legend:

> - next step

* - operations in a pop-up window

# - a comment

1. Launch Fiji Software
2. File > Open # open a micro-CT image - read into RAM (File > Import > TIFF Virtual Stack – read an image directly from drive storage)
3. Image > Type > 8-bit # convert image to 8-bit type
4. Image > Crop # resize and crop the image
5. Image > Adjust > Brightness/Contrast # adjust brightness and contrast
6. Plugins > BaSiC # apply a X-ray attenuation correction
7. File > Save As > Tiff # save the corrected image
8. Image > Adjust > Threshold > * (check stack histogram) > Apply > * (uncheck calculate threshold for each image) # choose and extract chosen components to a binary image format at appropriate threshold level
9. Plugins > 3D > 3D Fast Filters > * median # apply 3D median filter on the binary image
10. Plugins > MorphoLibJ > Kill Borders # remove objects at the edges of the image
11. Plugins > MorphoLibJ > Binary Images > Size opening 2D/3D # remove objects below chosen size
12. Plugins > 3D > 3D Simple Segmentation # assign ID to each separate object
13. File > Save As > Tiff # save the segmented image
14. Plugins > MorphoLibJ > Analyze > Analyze Regions 3D > * File > Save As > regions.csv # perform and save the measurements; the segmented image is required
15. Plugins > MorphoLibJ > Analyze > Equivalent Ellipsoid > * (eigenvectors table) File > Save As > eigenvectors.csv # perform and save the measurements; opened the segmented image is required
16. Plugins > MorphoLibJ > Analyze > Geodesic Diameter 3D > * File > Save As > geodesic.csv # perform and save the measurements; the segmented and corrected images are required
17. Plugins > MorphoLibJ > Analyze > Intensity Measurements 2D/3D > * File > Save As > intensity.csv # perform and save measurements, opened the segmented and corrected images are required
18. Plugins > 3D > 3D Manager > * Settings > * (in the measurements section check all but Convex Hull) > OK > Add Image > Select All > Measure 3D * File > Save As > measure.csv # perform and save the measurements; the segmented image is required
19. Plugins > MorphoLibJ > Binary Images > Assign Measure to Label # assign values from a table to the objects in the segmented image i.e., cluster id. (Open > clusters.csv)
20. Plugins > 3D Viewer # visualize the image in 3D

## Appendix C. Data Description and Manipulation

**Table A1:** The description of the object parameters resulting from the image analysis based on [45,46] and modified by the authors.

Category	No.	Parameter	Description
Chemical Composition	1	mean intensity	mean of voxel values in the object
	2	intensity SD	standard deviations of voxel values in the object
	3	max intensity	maximum of voxel value in the object
	4	min intensity	minimum of voxel value in the object
	5	median intensity	median of voxel values in the object
	6	mode intensity	mode of voxel values in the object
	7	intensity skewness	the skewness of voxel values distribution in the object
	8	intensity kurtosis	kurtosis of voxel values distribution in the object
Size	9	object volume	the volume of the object
	10	ellipsoid volume	the volume of the ellipsoid fitted to the object
	11	box volume	the volume of the bounding box encompassing the object
	12	ball volume	the volume of the largest ball inscribed in the object
	13	surface area	the sum of the surface area of contouring voxel faces
	14	Feret diameter	the largest calibrated distance between two contour voxels in the object
	15	mean breadth	the mean of the Feret diameter measured in all directions of the object
	16	geodesic diameter	the length of the longest geodesic path within the object
	17	ball radius	the radius of the largest inscribed ball
	18	semi-axis a	the length of the longest semi-axis of the ellipsoid
	19	semi-axis b	the length of the intermediate semi-axis of the ellipsoid
	20	semi-axis c	the length of the shortest semi-axis of the ellipsoid
	21	mean radius	mean distance from the geometrical centre of the object to the surface
	22	radius SD	the standard deviation of distance from the geometrical centre of the object to surface
	23	max radius	maximum distance from the geometrical centre of the object to the surface
	24	min radius	minimum distance from the geometrical centre of the object to the surface
Shape	25	compactness	the normalized ratio between the surface and the volume of the object
	26	discrete compactness	the measure of compactness for porous and fragmented objects basing on the face-connectivity of voxels within the object
	27	sphericity	root square of the compactness
	28	Euler number	the number of connected fragments of the object, minus the number of holes in the object, plus the number of bubbles within it
	29	geodesic elongation	the ratio between the geodesic diameter and the diameter of the largest inscribed ball
	30	semi-axes ratio a/b	the ratio between the length of the largest and medium semi-axis of the ellipsoid
	31	semi-axes ratio a/c	the ratio between the lengths of the largest and smallest semi-axis of the ellipsoid
	32	semi-axes ratio b/c	the ratio between the length of the medium and smallest semi-axis of the ellipsoid
	33	object vol./ellipsoid vol. ratio	the ratio between the volume of the object and the ellipsoid
	34	object vol./box vol. ratio	the ratio between the volume of the object and the bounding box
	35	ball vol./object vol. ratio	the ratio between the volume of the ball and the object
Spatial Arrangement and Orientation	36	x coordinate	x coordinate of the geometrical centre for each object
	37	y coordinate	y coordinate of the geometrical centre for each object
	38	z coordinate	z coordinate of the geometrical centre for each object
	39	semi-axis a trend	the direction of the ellipsoid’s semi-axis a on the xy plane measured clockwise from the north
	40	semi-axis a plunge	the vertical angle, measured downwards, between the xy plane and the ellipsoid’s semi-axis a
	41	semi-axis b trend	the direction of the ellipsoid’s semi-axis b on the xy plane measured clockwise from the north
	42	semi-axis b plunge	the vertical angle, measured downwards, between the xy plane and the ellipsoid’s semi-axis b
	43	semi-axis c trend	the direction of the ellipsoid’s semi-axis c on the xy plane measured clockwise from the north
	44	semi-axis c plunge	the vertical angle, measured downwards, between the xy plane and the ellipsoid’s semi-axis c

**Table A2:** The libraries utilised for data manipulation, visualization and analysis in R.

Library Name and Version	Usage	Reference
base_4.0.3	data manipulation	[47]
openair_2.8-1	data visualization	[48]
ggpmisc_0.3.7	data manipulation	[49]
cluster_2.1.0	cluster analysis	[50]
viridis_0.5.1	data visualization	[51]
Morpho_2.8	data manipulation	[52]
ggrepel_0.9.0	data visualization	[53]
gridExtra_2.3	data visualization	[54]
tidyverse_1.3.0	data manipulation	[55]
reshape2_1.4.4	data manipulation	[56]
factoextra_1.0.7	cluster analysis	[57]
qgraph_1.6.5	data visualization	[58]
ggplot2_3.3.3	data visualization	[59]
corrplot_0.84	data visualization	[60]
psych_2.0.12	data exploration	[61]
DescTools_0.99.39	data manipulation	[62]

## Appendix E. The Protocol Validation on a Standard Sample

The validity of measurements and computations was verified on a standard sample. The sample was built in the Fiji software (the macro for the standard sample creation in Fiji is available at https://github.com/piotrstrzelecki/materials, accessed on 5 June 2021) and consists of 180 artificially generated grains of known properties in the image of 700 × 700 × 600 pixel in size. Each grain’s centre is equidistant (100 pixels) from the closest neighbours. The sample is composed of five layers and each layer consists of 36 grains. Within each layer, grains have the same shape. The uppermost layer consists of spherical grains. Toward the bottom of the sample, the shape of grains changes and grains acquire more ellipsoidal up to discoidal shapes. The lowermost layer consists of rod-shaped grains (Figure A2a). Each object has unique properties. The properties gradually change with increasing x, y and z coordinates of objects. With increasing y coordinate, the length of objects’ semi-axis a increases from 5 to 45 pixels and its trend increases from 0 to 330° in a 60° step. With increasing x coordinate, the plunge of grains’ semi-axis a increases from 0 to 90°.

The artificial grains were clustered into 3 groups based on the parameters resulting from image analysis and calculations (Figure A2b). The first cluster is composed of the biggest grains of spherical and ellipsoidal shapes (Figure A3a; the highest values of all semi-axes), whereas the second one consists of the smallest grains of regular shape (Figure A3a; the lowest values of semi-axes a and b). The third cluster is composed of discoidal and rod-shaped grains. shape (Figure A3a; the lowest values of semi-axes c). The grains belonging to the first and second clusters are present near the geometrical centre of the sample, whereas the grains of the third cluster are distant from the sample’s centre (Figure A2b, Figure A3b). The trends of semi-axis a in the first and second clusters are similar as the grains within these clusters occupy similar xy position in the sample and have larger angles in comparison to the grains in the second cluster, which are localised closer to the origin of the xy coordinate system (Figure A2c). The plunge angles are diversified in all clusters and show values from 0 to 90°. The expected properties find confirmation in the numerical data, hence confirming the correctness of the calculations.
